# Eosinophil count combined with routine indicators enhances early risk prediction value for ARDS in ICU patients: a retrospective cohort study

**DOI:** 10.1515/med-2026-1401

**Published:** 2026-03-18

**Authors:** WeiNing Ma, Hua Gao, MingZhe Wen

**Affiliations:** Department of Pediatrics, Shanghai General Hospital, Shanghai, China; Department of Gynaecology and Obstetrics, Shanghai General Hospital, Shanghai, China; Department of Interventional Therapy, Shanghai Ninth People’s Hospital, Shanghai Jiao Tong University School of Medicine, Shanghai, China

**Keywords:** acute respiratory distress syndrome, eosinopenia, intensive care unit, risk prediction, retrospective cohort study

## Abstract

**Objectives:**

This study aims to systematically evaluate the incremental value of admission eosinopenia EOS(−) in enhancing the predictive efficacy of early-stage acute respiratory distress syndrome (ARDS) risk models for ICU patients.

**Methods:**

This study employed a single-center retrospective cohort design, enrolling 482 adult ICU patients between 2020 and 2023. Using Cox proportional hazards regression, we constructed and compared three models: Model 1 (baseline clinical model) included age, pneumonia, sepsis, and SOFA score; Model 2 added lymphocytes and eosinophils to Model 1; Model 3 further adjusted for BMI and diabetes. Predictive performance was assessed using the C-index, net reclassification improvement (NRI), and integrated discrimination improvement (IDI), and validated internally.

**Results:**

C-statistics for Models 1, 2, and 3 were 0.683, 0.710, and 0.729, respectively. Model 3 demonstrated optimal performance (NRI=0.185, p=0.004), with an adjusted C-statistic of 0.695. Multivariate analysis identified age, pneumonia, and sepsis as independent ARDS risk factors, while EOS(−) showed non-independence. Results from competing risks analysis were consistent with the primary analysis conclusions.

**Conclusions:**

Although eosinopenia is not an independent predictor of ARDS, it provides incremental information for early risk stratification based on conventional clinical factors, thereby aiding in the identification of high-risk patients.

## Introduction

Acute respiratory distress syndrome (ARDS) remains a critical condition in intensive care unit (ICU), with its high incidence and mortality rates consistently attracting clinical attention [1]. The incidence of ARDS in ICUs ranges from 10 % to 20 %. Despite continuous optimization of mechanical ventilation strategies and supportive therapies, in-hospital mortality remains as high as 35–40 % [2], 3]. The pathophysiological basis of ARDS involves disruption of the alveolar-capillary barrier and uncontrolled systemic inflammatory responses. Precise identification of high-risk populations and early intervention targeting inflammatory networks are critical for improving outcomes [4]. Currently, commonly used clinical risk prediction tools (e.g., APACHE II score, SOFA score) can quantify overall disease severity through multi-organ function assessment [5], 6]. However, as a comprehensive assessment tool, the core limitation of such scoring systems lies in their inability to specifically reflect the immune-inflammatory dysregulation pathways that drive ARDS.

With the deepening exploration of ARDS pathophysiological mechanisms – such as damage to the alveolar-capillary barrier, dysregulated immune inflammation, and epithelial/endothelial cell necrosis – a large number of biomarkers targeting specific pathophysiological pathways have been reported. For example, soluble receptor for advanced glycation end products [7], reflecting lung epithelial cell injury, and clara cell protein 16 [8], angiopoietin-2 [9] indicating vascular endothelial dysfunction, and interleukin [10] involved in the inflammatory cascade reaction, have all been confirmed to be closely associated with the development and prognosis of ARDS. However, most existing biomarkers still face significant clinical translation bottlenecks: their detection limitations are pronounced, with some markers (such as the protein ratio in alveolar fluid) requiring invasive sampling or time-consuming testing cycles, making them unsuitable for rapid bedside decision-making. Additionally, clinical acceptance remains low – no single biomarker or biomarker combination has yet been incorporated into internationally recommended routine assessment systems. More critically, existing research primarily focuses on molecular markers associated with inflammatory injury or barrier disruption. Systematic exploration and predictive value assessment of cellular immune markers – such as circulating immune cell subsets – that target the core pathological mechanism of “immune dysregulation” and can be obtained instantly through routine blood tests remain insufficient.

As a key component of innate immune cells, changes in eosinophil counts during infections, allergies, and autoimmune diseases have been extensively studied [11], [12], [13]. In critically ill patients, hypovolemia (peripheral blood count <0.05 × 10^9^/L) [14] is regarded as a marker of stress response or immunosuppression, with mechanisms potentially involving glucocorticoid release, cytokine storm-induced granulocyte migration, or apoptosis [15], [16], [17]. However, the role of eosinophils in the pathogenesis of ARDS remains controversial. On one hand, eosinophils may contribute to lung tissue repair by releasing anti-inflammatory factors (e.g., IL-10) and modulating Th2-type immune responses; their depletion could potentially weaken the host’s anti-inflammatory capacity. On the other hand, eosinopenia may also represent a concomitant manifestation of systemic inflammatory imbalance, reflecting disease severity rather than serving as a direct pathogenic factor [18].

Based on the above background, this study proposes the following core hypothesis: Eosinopenia at admission significantly enhances the discriminatory and reclassification capabilities of ARDS risk prediction models based on conventional clinical indicators (such as age, specific infections, and organ function scores). To validate this hypothesis, this study employs a single-center retrospective cohort design. By constructing and comparing a series of predictive models, it systematically evaluates the extent to which hypovolemia enhances model discrimination and reclassification capabilities. The aim is to clarify its clinical utility and role in ARDS risk stratification, providing evidence-based support for developing more efficient and practical bedside risk assessment tools.

## Materials and methods

### Study population

This study was a single-center, retrospective cohort study utilizing data from Shanghai General Hospital’s database. This retrospective cohort study was conducted in accordance with the ethical standards of the institutional review board and the Declaration of Helsinki. The study protocol was reviewed and approved by the Ethics Committee of Shanghai General Hospital. Due to the anonymity and retrospective nature of the data analysis, informed consent was waived. The study population consisted of adult patients (aged≥18 years) who were admitted to the ICU for the first time between January 2020 and December 2023. Inclusion criteria included: 1) First admission to the ICU; 2) A complete blood count (CBC) test record within 24 h of ICU admission, which must include an eosinophil count. Exclusion criteria were: 1) ICU stay duration <48 h (to ensure sufficient time for ARDS development); 2) ARDS diagnosis at admission or within 24 h of ICU admission (to ensure exposure preceded outcome); 3) Presence of underlying conditions known to cause persistent elevation of eosinophils, such as eosinophilic pneumonia, asthma, eosinophilic granulomatosis with polyangiitis, active parasitic infections, or specific hematologic malignancies, to control for confounding factors; 4) Severe deficiencies in medical records, such as missing core indicators required for APACHE II scoring.

### Grouping and outcome definition

In the exposure variables and grouping settings, the eosinophil count in the first venous blood sample collected after ICU admission served as the core indicator. Based on evidence-based medical evidence and clinical consensus, and in conjunction with relevant literature [19], patients were divided into two groups: the eosinopenia group (EOS(−), <0.05 × 10^9^/L) and a non-eosinopenia group (Non EOS(−), ≥0.05 × 10^9^/L).

According to the Berlin criteria for ARDS [20], the outcome was defined, with the first time these criteria were met after ICU admission marking the endpoint event. Diagnosis required simultaneous fulfillment of four core criteria: onset timing requires symptoms appearing within one week of known clinical injury or new/worsening respiratory manifestations; chest imaging shows bilateral patchy opacities with exclusion of alternative explanations such as pleural effusion, atelectasis, or nodules; the origin of edema must be assessed through medical records and echocardiography to exclude cardiac causes; oxygenation status is graded based on the PaO_2_/FiO_2_ ratio: mild (200<PaO_2_/FiO_2_≤300 mmHg and PEEP/CPAP≥5 cmH_2_O), moderate (100<PaO_2_/FiO_2_≤200 mmHg and PEEP/CPAP≥5 cmH_2_O), and severe (PaO_2_/FiO_2_≤100 mmHg and PEEP/CPAP≥5 cmH_2_O). By automatically extracting blood gas analysis data and integrating it with physician diagnostic records and chest X-ray reports, two independent investigators conducted blinded review and confirmation of diagnoses to ensure standardization and reproducibility of the diagnostic process. This rigorous system for variable definition and outcome assessment laid a solid foundation for subsequent causal inference and confounding control. Two investigators, unaware of the patients’ eosinophil counts (blinded), independently reviewed all chest imaging (radiography or CT) materials and corresponding reports for these patients. For cases with diagnostic discrepancies, a third senior critical care medicine specialist was invited to arbitrate and reach a final diagnostic consensus. Diagnostic agreement between the two investigators was assessed using Cohen’s Kappa coefficient (Kappa=0.81). Cases with diagnostic discrepancies were resolved by inviting a third senior critical care medicine specialist to arbitrate and reach a final diagnosis. This process ensured the objectivity and standardization of all ARDS diagnoses.

### Data collection and processing

All data were extracted through the electronic medical record review system. The scope of data extraction encompassed all available records from the patient’s admission and ICU stay. To ensure clarity in variable definitions and provide an accurate framework for subsequent analysis, variables were systematically categorized into three groups based on their clinical significance and temporal logic for collection and delineation.

Variables reflecting patients’ baseline characteristics were collected, which were determined at or prior to ICU admission. These included demographic information (age, gender, admission type, height and weight for BMI calculation) and underlying comorbidities identified through ICD codes (e.g., hypertension, diabetes). Variables reflecting the patient’s acute condition at admission were defined, encompassing the direct cause of ICU admission (e.g., sepsis, pneumonia), admission type, APACHE II and SOFA scores quantifying initial severity. All components of the SOFA score (respiratory, coagulation, hepatic, cardiovascular, neurological, renal) are calculated strictly based on the worst physiological indicator or laboratory test result within 24 h of ICU admission. Specific data (such as PaO_2_/FiO_2_, platelet count, bilirubin, vasoactive drug dosage and duration, Glasgow Coma Scale score, creatinine, or urine output) are directly extracted and verified from intensive care unit (ICU) records, anesthesia logs, and laboratory databases. The eosinophil count at admission (measured as the absolute value in the first venous blood sample collected within 6 h of ICU admission). Major therapeutic interventions within the first 24 h of ICU admission, including mechanical ventilation, glucocorticoid administration, and vasoactive drug use, were documented. These measures reflect disease severity and may directly influence subsequent clinical outcomes.

According to the Berlin criteria, the primary outcome, ARDS was strictly identified, confirmed by automated parameter extraction and manual review, and was required to happen after eosinopenia was first detected. For missing data, a staged strategy was applied: cases with key variable missing rates >15 % were excluded; continuous variables with <5 % missing rates underwent multiple imputation; categorical variables were coded as “unknown.” All primary analyses were based on this processed dataset.

### Sample size and statistical power analysis

This study is an exploratory retrospective cohort study that ultimately included 482 patients, among whom 108 (22.4 %) experienced the primary outcome event (ARDS). In statistical analysis, to ensure robustness of the multivariate model and avoid overfitting, variable selection and model construction followed the empirical principle of Events Per Variable (EPV) in predictive modeling studies, ensuring EPV ≥10 in core analyses. In the final full-variable predictive model, we plan to include no more than 10 core predictive variables. Accordingly, the calculated EPV for this study is approximately 10.8, meeting the minimum requirement of this empirical principle. This indicates that the current sample size and number of events are sufficient to support the planned model construction and subsequent analyses.

Based on the anticipated sample size and estimated outcome event rate (referencing prior literature and our center’s historical data), this study will possess sufficient statistical power (≥80 %, α=0.05, two-tailed test) to identify statistically significant incremental predictive value.

### Statistical analysis

All statistical analyses were performed using R software (version 4.3.1). A two-tailed p<0.05 was considered statistically significant. Continuous variables were expressed as mean ± standard deviation for those meeting normality criteria, with independent samples *t*-tests used for intergroup comparisons. Variables not meeting normality criteria were expressed as median (interquartile range, IQR), with Mann-Whitney U tests used for intergroup comparisons. Categorical variables were expressed as case counts (percentages), with chi-square tests or Fisher’s exact tests used for intergroup comparisons. To construct a robust multivariate model, the variance inflation factor (VIF) was first calculated for all candidate predictor variables to assess multicollinearity, with VIF <5 considered acceptable. Subsequently, the random forest algorithm was employed to rank variable importance, aiding variable selection in the subsequent model. The dataset was randomly split into a training set (n=337) and a validation set (n=145) at a 7:3 ratio. Three nested Cox proportional hazards regression models were constructed on the training set: Model 1 (basic clinical model) included age, pneumonia, sepsis, and SOFA score; Model 2 added lymphocyte count and eosinophil status to Model 1; Model 3 further incorporated BMI and diabetes. On the training set, the C-index (consistency index) was used to evaluate model discriminative ability, and the likelihood ratio test was applied to compare the goodness-of-fit among nested models. The predictive performance gains from new indicators were quantified by calculating the net reclassification improvement (NRI) and integrated discrimination improvement (IDI). The C-index of the aforementioned models was calculated on the validation set. Internal validation was performed on the optimal model obtained from the training set using the Bootstrap method (1,000 repetitions) to obtain a bias-corrected C-index, thereby assessing the model’s generalization ability. Based on the optimal model, a column chart was plotted to visualize individualized risk. Calibration curves were used to assess the consistency between the model’s predicted probabilities and the actual observed risks. Decision curve analysis (DCA) evaluated the clinical net benefit of the model at different threshold probabilities to assess its clinical utility. Survival curves were plotted using the Kaplan-Meier method, and group differences were analyzed using the log-rank test. The relationship between EOS status and ARDS risk was explored. To assess potential bias from mortality as a competing event affecting the primary outcome (ARDS occurrence), competing risks analysis was supplemented in this study. “In-hospital death” was defined as the competing event. The Fine-Gray proportional hazards model was used to calculate subdistribution hazard ratios (sHR) and their 95 % confidence intervals, evaluating the association between each predictor variable and ARDS risk after accounting for competing risks.

### Ethics statement

The present study was approved by the Ethics Committee of Shanghai General Hospital. All procedures were performed in accordance with the ethical standards of the Institutional Review Board and The Declaration of Helsinki, and its later amendments or comparable ethical standards.

### Informed consent

As it is a retrospective observational study and all analyzed data are anonymized, posing no additional risk to patients, the requirement for informed consent was waived.

## Results

### Baseline characteristics

As shown in Table 1, this study included 482 ICU patients, of whom 108 (22.4 %) developed ARDS during hospitalization (ARDS group), while the remaining 374 (77.6 %) did not develop ARDS (non-ARDS group). Comparison of baseline characteristics revealed that patients in the ARDS group were older [median: 73 years vs. 64 years, p<0.001], lower BMI [23.5 kg/m^2^ vs. 24.8 kg/m^2^, p=0.011], and a higher prevalence of diabetes (34.3 % vs. 18.2 %, p<0.001). Regarding disease severity, the ARDS group had higher APACHE II scores [21.0 vs. 19.0, p=0.002], along with higher rates of sepsis (63.9 % vs. 44.9 %, p<0.001) and pneumonia (46.3 % vs. 27.5 %, p<0.001). While SOFA scores and hypertension prevalence showed no significant differences between groups (both p>0.05). Therapeutically, the ARDS group received mechanical ventilation (73.1 % vs. 62.0 %, p=0.033) and glucocorticoid therapy (24.1 % vs. 14.7 %, p=0.022) at a higher rate within 24 h of admission. Among laboratory findings, the most prominent difference was a significantly lower eosinophil count in the ARDS group [0.03 × 10^9^/L vs. 0.11 × 10^9^/L, p=0.002], while white blood cell and lymphocyte counts showed no statistically significant differences between groups (both p>0.05).

**Table 1:** Baseline characteristics of study population.

Characteristic	Non-ARDS n=374	ARDS n=108	p-Value
Demographics and baseline characteristics			
Age, median (Q1, Q3)	64 (57, 71)	73 (62, 79)	<0.001
Gender, n (%)			0.456
Female	141 (37.7 %)	45 (41.7 %)	
Male	233 (62.3 %)	63 (58.3 %)	
BMI, kg/m^2^, median (Q1, Q3)	24.8 (22.2, 27.3)	23.5 (20.9, 26.6)	0.011
Condition and diagnosis at admission			
APACHE Ⅱ, median (Q1, Q3)	19.0 (15.0, 23.0)	21.0 (17.0, 24.0)	0.002
SOFA rating, median (Q1, Q3)	6.00 (4.00, 8.00)	6.00 (4.00, 8.00)	0.879
Sepsis, n (%)	168 (44.9 %)	69 (63.9 %)	<0.001
Pneumonia, n (%)	103 (27.5 %)	50 (46.3 %)	<0.001
Hypertension, n (%)	156 (41.7 %)	52 (48.1 %)	0.234
Diabetes, n (%)	68 (18.2 %)	37 (34.3 %)	<0.001
Intervention within 24 h of admission			
Mechanical ventilation, n (%)	232 (62.0 %)	79 (73.1 %)	0.033
Glucocorticoid administration, n (%)	55 (14.7 %)	26 (24.1 %)	0.022
EOS, ×10^9^/L, M (Q_1_, Q_3_)	0.11 (0.02, 0.23)	0.03 (0.02, 0.18)	0.002
WBC, ×10^9^/L, M (Q_1_, Q_3_)	12.2 (9.4, 15.2)	12.9 (10.0, 16.4)	0.114
LYM, ×10^9^/L, M (Q_1_, Q_3_)	0.90 (0.70, 1.20)	0.90 (0.70, 1.10)	0.148

Non-normally distributed variables were expressed as median (interquartile range) and analyzed using the Mann-Whitney U test. Categorical variables were expressed as n (%) and analyzed using chi-square tests. BMI, body mass index; APACHE, acute physiology and chronic health evaluation; SOFA, sequential organ failure assessment; EOS, eosinophil count; WBC, white blood cell count; LYM, lymphocyte count. Severity at admission was quantified using APACHE II and SOFA scores (based on the worst indicator within 24 h of admission) and the primary diagnosis for this admission (e.g., Sepsis, Pneumonia). Early interventions (mechanical ventilation and glucocorticoid administration) were defined as therapeutic measures initiated within 24 h of admission; laboratory parameters were defined as the first recorded value within 6 h of admission.

### Primary and secondary outcomes

As shown in Table 2, among patients with ARDS, a significantly higher proportion exhibited eosinopenia (EOS(−)): 55.56 % vs. 37.97 % (p=0.001). Furthermore, ICU length of stay was markedly longer in the ARDS group [(14.5 ± 3.9) days vs. (6.3 ± 2.7) days, p<0.001], and in-hospital mortality was significantly higher compared to the non-ARDS group (30.6 % vs. 19.0 %, p=0.010).

**Table 2:** Comparison of eosinopenia status distribution and clinical outcomes between ARDS and non-ARDS patients.

Characteristic	Non-ARDS n=374	ARDS n=108	p-Value
EOS status			0.001
EOS (−)	142 (37.97 %)	60 (55.56 %)	
Non EOS (−)	232 (62.03 %)	48 (44.44 %)	
ICU days, mean ± SD	6.3 ± 2.7	14.5 ± 3.9	<0.001
In-hospital death, n (%)	71 (19.0 %)	33 (30.6 %)	0.010

Non-normally distributed variables were expressed as median (interquartile range) and analyzed using the Mann-Whitney U test. Categorical variables were expressed as n (%) and analyzed using the chi-square test.

### Construction and comparison of multivariate Cox regression models

**Figure 1:**
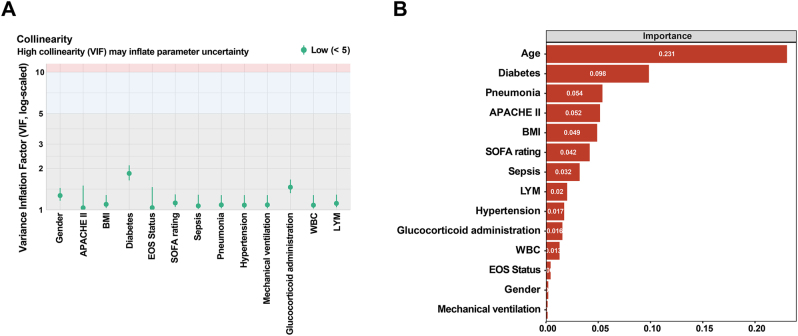
Collinearity analysis and importance ranking of predictor variables. (A) Demonstrates the collinearity among candidate predictor variables. The variance inflation factors (VIF) are all below 5, indicating no significant multicollinearity among variables, making them suitable for inclusion in a multivariate model. (B) Presents the importance ranking of each variable for ARDS occurrence, assessed using the random forest algorithm. BMI, body mass index; APACHE, acute physiology and chronic health evaluation; SOFA, sequential organ failure assessment; EOS, eosinophil count; WBC, white blood cell count; LYM, lymphocyte count.

To construct a robust predictive model and avoid overfitting, we first screened candidate predictor variables. VIF testing revealed no significant multicollinearity among variables (VIF <5, Figure 1A), indicating relatively independent information contribution. Given the limited number of outcome events in this study (n=108), we further employed a random forest algorithm to assess variable importance, guiding the prioritization of core variables for subsequent models (Figure 1B).

**Figure 2:**
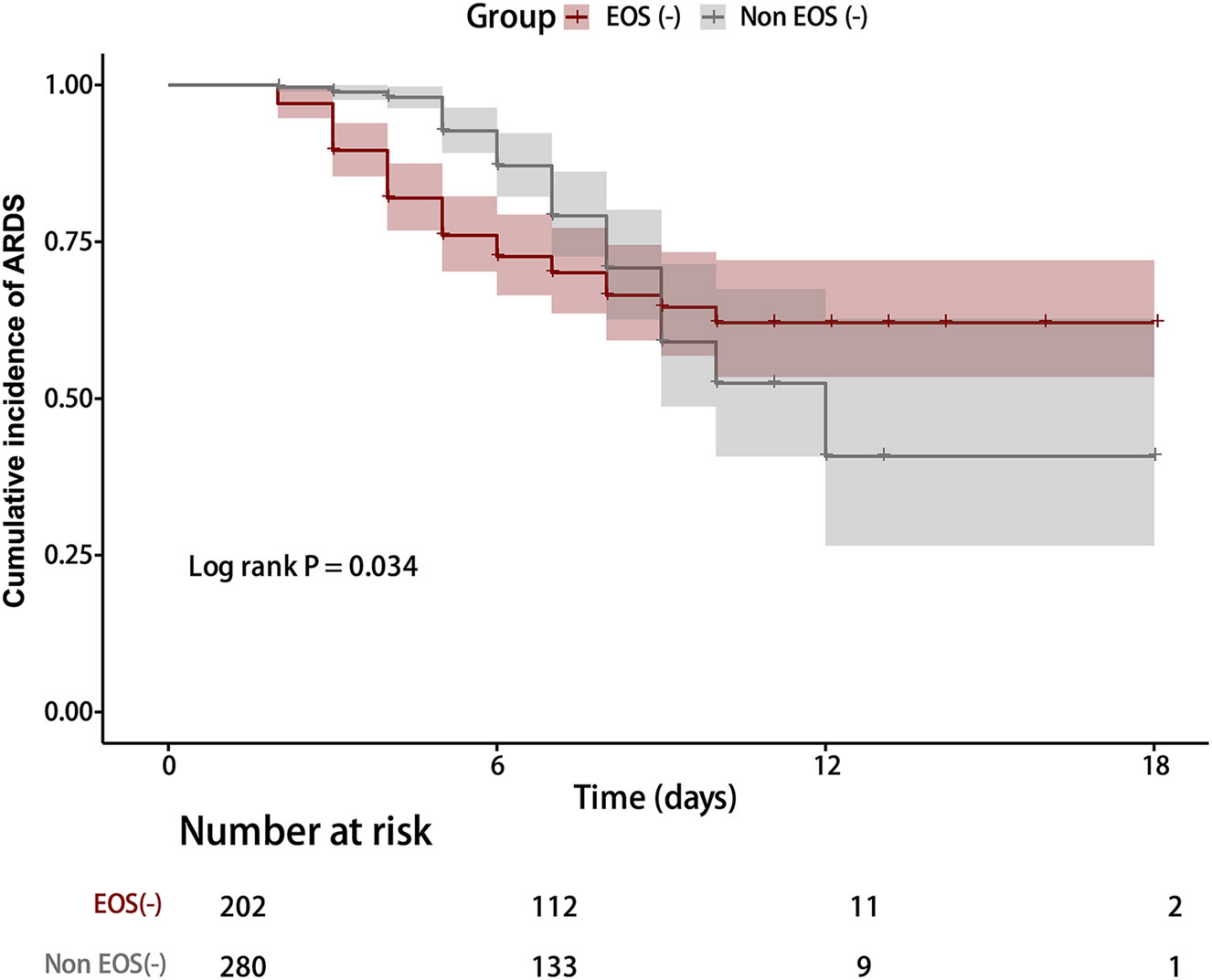
Cumulative incidence curve of ARDS. The Kaplan-Meier curve shows the cumulative incidence of ARDS in ICU patients stratified by eosinophil count. The log-rank test confirmed a statistically significant difference between the two groups (p=0.034).

Based on the above analysis and clinical consensus, we constructed three progressively expanded Cox proportional hazards regression models to systematically evaluate the incremental predictive value of immune-inflammatory markers. Model 1 (baseline clinical model) included only age, pneumonia, sepsis, and SOFA score, yielding a predictive performance (c-index) of 0.683. After incorporating lymphocyte count and hypovolemic state into Model 1, Model 2’s c-index improved to 0.710, with statistically significant predictive enhancement (Net Rank Improvement Index NRI=0.152, p=0.014). After further adjusting for BMI and diabetes, Model 3 achieved a predictive performance of 0.729 (NRI relative to Model 1=0.185, p=0.004). Internal validation using the Bootstrap method yielded an adjusted C-index of 0.705 for Model 3, indicating robust stability (Table 3). Notably, although LYM and EOS did not demonstrate statistical independence in the multivariate model, their inclusion yielded sustained and measurable improvements in predictive performance, suggesting these readily accessible immune-inflammatory markers provide valuable supplementary information to conventional clinical risk assessment. Age, pneumonia, and sepsis remained consistent independent risk factors for ARDS across all three models. Furthermore, Kaplan-Meier analysis revealed a significantly higher cumulative ARDS incidence in the EOS(−) group compared to the Non EOS(−) group (log-rank test p=0.034, Figure 2).

**Table 3:** Comparison of Cox proportional hazards regression models for ARDS risk prediction.

Characteristic	Model 1		Model 2		Model 3	
	HR (95 % CI)	p-Value	HR (95 % CI)	p-Value	HR (95 % CI)	p-Value
Age, years	1.05 (1.03, 1.07)	<0.001	1.05 (1.03, 1.07)	<0.001	1.05 (1.03, 1.07)	<0.001
Pneumonia (yes)	2.04 (1.39, 2.98)	<0.001	1.92 (1.30, 2.83)	<0.001	1.94 (1.31, 2.86)	<0.001
Sepsis (yes)	1.72 (1.16, 2.56)	0.007	1.63 (1.09, 2.44)	0.017	1.62 (1.09, 2.42)	0.017
SOFA rating	0.96 (0.89, 1.03)	0.218	0.92 (0.85, 1.00)	0.054	0.92 (0.84, 1.00)	0.044
LYM, ×10^9^/L	–	–	0.73 (0.40, 1.33)	0.303	0.73 (0.43, 1.6)	0.416
EOS status (EOS(−))	–	–	0.69 (0.44, 1.09)	0.115	0.69 (0.43, 1.08)	0.107
BMI, kg/m^2^	–	–	–	–	0.94 (0.89, 0.99)	0.026
Diabetes (yes)	–	–	–	–	1.52 (1.01, 2.29)	0.043

**Key model info**						

N (total)	482		482		482	
Events	108		108		108	
C-index	0.683		0.710		0.729	
Log-likelihood	−569		−567		−562	

CI, confidence interval; HR, hazard ratio.

### Validation based on optimal prediction models

**Figure 3:**
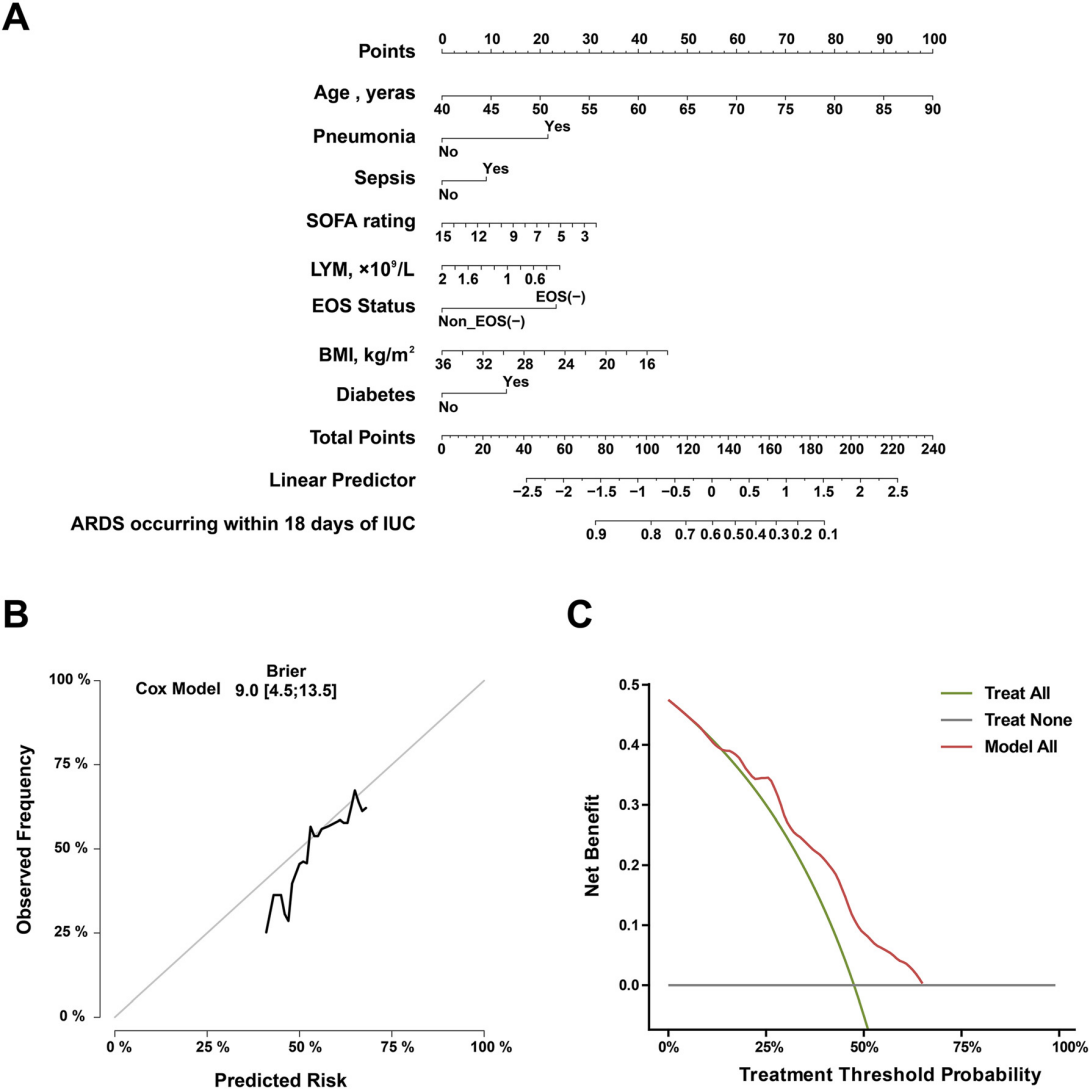
Clinical translation and validation based on the optimal predictive model. (A) Column chart for predicting ARDS risk in ICU patients: this column chart, constructed using a multivariate Cox proportional hazards model (Model 3), enables individualized prediction of the probability of ARDS occurrence within 18 days of ICU admission. The scale above each indicator corresponds to its score. Summing the scores of all items yields a total score, which can be used to read the corresponding ARDS probability on the risk scale below.

To facilitate the clinical translation of predictive models, we developed a visual nomogram based on the optimal-performance Model 3 (Figure 3A). This tool integrates eight indicators – age, pneumonia, sepsis, SOFA score, lymphocyte count, eosinophil status, BMI, and diabetes – enabling convenient individualized assessment of the absolute risk of ARDS occurrence within 18 days among ICU patients. The model calibration curve demonstrates good agreement between predicted and observed risks (Figure 3B). Further decision curve analysis indicates that risk stratification using this model and implementing targeted interventions within a reasonable decision threshold range of 5–50 % yields higher clinical net benefit than either “all intervention” or “no intervention” strategies (Figure 3C), confirming its potential clinical utility.

## Discussion

This study aimed to evaluate whether hypovolemia provides incremental value for early risk stratification of ARDS in ICU patients. We found that: 1) In unadjusted analyses, hypovolemia was associated with higher ARDS risk, mortality, and longer ICU length of stay; 2) In predictive modeling, a baseline clinical model comprising age, pneumonia, sepsis, and SOFA score demonstrated moderate predictive performance (C-index=0.683). Incorporating lymphocyte count and hypovolemic status significantly enhanced the model’s discriminatory and reclassification capabilities (C-index=0.710, NRI=0.152, p=0.014); 3) However, in both multivariate Cox and competing risks models, eosinopenia itself did not demonstrate statistical independence, whereas age, pneumonia, and sepsis were confirmed as stable and strongest independent risk factors.

This study further confirms that age, pneumonia, and sepsis are stable and independent risk factors for ARDS development. This finding aligns with numerous previous studies, reaffirming the central role of infection and advanced age in ARDS pathogenesis. In contrast, although lymphopenia and eosinopenia were both associated with high risk in univariate analysis, neither demonstrated statistical independence in multivariate models. This result was further validated by competing risks analysis: even after accounting for in-hospital death as a competing event, the association between eosinopenia and ARDS remained non-independent (sHR=1.44, 95 % CI: 0.91–2.29, p=0.121; Supplementary Table 1). Collectively, these findings suggest that lymphopenia and hypovolemia more likely reflect a common systemic stress and immunosuppression state in critically ill patients – particularly those with severe infection – rather than being specific drivers of ARDS.

This interpretation aligns with current understanding of critical illness pathophysiology. Eosinophils exhibit high sensitivity to endogenous cortisol. Under conditions of severe stress, infection, or systemic inflammatory response, activation of the hypothalamic-pituitary-adrenal (HPA) axis can induce their apoptosis or redistribution [21], [22], [23]. Therefore, the eosinopenia observed in this study is likely a downstream manifestation of high levels of inflammation and stress in patients (driven by sepsis, pneumonia, etc.). After controlling for these underlying drivers in multivariate models, its seemingly “independent” predictive role is superseded. This finding aligns with previous research concluding that while eosinopenia frequently accompanies poor outcomes in patients with sepsis or pneumonia, its impact is primarily attributable to the underlying severity of the disease [24], 25]. The incremental value of this study lies in objectively demonstrating, through model comparisons and NRI quantification, that even as a “concomitant biomarker,” the combination of eosinopenia and lymphocyte count provides statistically significant incremental information to risk assessment based on conventional clinical factors.

Multivariate analysis results from this study indicate that age, pneumonia, and sepsis are independent risk factors for ARDS in ICU patients. This finding aligns with existing clinical understanding and previous research conclusions, further solidifying the core clinical basis for ARDS risk assessment. Patients in the ARDS group were older and had higher SOFA scores, factors that inherently increase ARDS risk [26], 27]. Aging is often accompanied by a physiological decline in immune function and reduced pulmonary reserve capacity [28]. Sepsis, as a key trigger for uncontrolled inflammatory responses, can directly damage the alveolar-capillary barrier [29]. Both factors are established drivers of ARDS development, underscoring the need for clinical focus on this high-risk population. Pneumonia, as an infectious disease directly affecting the lung parenchyma, involves pathogens (bacteria, viruses, etc.) that can cause direct damage to pulmonary epithelial cells and activate local inflammatory responses. This triggers alveolar cavity exudation and edema. If the inflammatory response becomes uncontrolled, it can progress to ARDS [30]. In clinical practice, such patients often require earlier initiation of intensive monitoring and intervention measures. The findings of this study reiterate the need for vigilance regarding the risk of ARDS in elderly ICU patients with concomitant pneumonia or sepsis, providing theoretical support for managing this critical clinical population. Notably, although hypovolemia did not emerge as an independent predictor of ARDS in multivariate analysis, this does not negate its clinical value. As a simple, rapid, cost-effective, and readily available laboratory parameter, the admission eosinophil count holds potential for bedside application. In the context of critical illness, where the body is under intense stress, massive glucocorticoid release and cytokine storms can induce granulocyte migration to inflammatory sites or apoptosis, both leading to peripheral blood eosinopenia. This change essentially represents an “integrative signal” of immune-inflammatory imbalance rather than a direct pathogenic factor driving ARDS development [31], 32]. A systematic review reported that 11 studies demonstrated a correlation between hypovolemia and sepsis, while 8 studies identified persistent hypovolemia associated with ICU admission beyond 48 h [31]. Therefore, while it cannot serve as an independent predictive indicator, it provides additional risk stratification information for clinical practice when combined with core risk factors such as age, pneumonia, and sepsis. Compared to biomarkers that rely on invasive sampling (e.g., bronchoalveolar lavage fluid testing) [33], are time-consuming, or are costly (e.g., soluble advanced glycation end-product receptors, angiopoietin-2, etc.) [34].

Some studies indicate that alterations in immune cell subsets are closely associated with the risk of ARDS development in critically ill patients [35]. In a propensity score-matched analysis, the 28-day mortality rate among ARDS patients with reduced EOS was significantly higher than that among patients without reduced EOS [36]. Although this study focused on predicting mortality outcomes rather than the risk of ARDS development, it confirmed the association between eosinopenia and disease severity in ARDS patients from a prognostic perspective. Furthermore, existing research lacked systematic evaluation of the incremental value of predictive models. By employing nested model design and multiple statistical validations, this study quantified the supplementary predictive value of eosinopenia status, thereby providing a valuable addition to the current literature. Notably, compared to biomarkers requiring invasive sampling or time-consuming testing, eosinophil counts derive from routine blood tests, offering advantages of low cost and easy accessibility. This makes them better suited for rapid bedside clinical decision-making.

This study has several limitations that should be considered when interpreting the results: First, as a single-center retrospective study, despite efforts to minimize bias through strict inclusion and exclusion criteria and multiple imputation methods, selection bias and the risk of incomplete data recording remain unavoidable. Second, this study analyzed eosinophil counts at a single time point upon admission. While this metric offers the advantages of early detection and accessibility, a single measurement fails to capture its dynamic trajectory throughout the ICU course (e.g., whether reflecting transient stress-induced decline or persistent immunosuppression). Consequently, it may inadequately reveal the temporal association and underlying mechanisms linking eosinophilia to ARDS development. Future prospective studies should incorporate systematic multi-time-point monitoring to clarify the predictive value and pathophysiological significance of eosinophil dynamic patterns. Third, despite adjusting for numerous important clinical confounders, residual confounding may persist, such as unmeasured inflammatory markers or subtle treatment variations. Finally, the external validity of this study requires validation across different geographic regions and healthcare centers of varying scales.

## Conclusions

Age, pneumonia, and sepsis are classic and independent risk factors. Building upon this foundation, hypovolemia at admission – a readily accessible bedside indicator – though not an independent predictor, provides significant incremental value to routine clinical assessment when combined with lymphocyte count to offer comprehensive immune-inflammatory information. This facilitates more refined early risk stratification.

## Supplementary Material

Supplementary Material
