# Supplementary material for: Eosinophil count combined with routine indicators enhances early risk prediction value for ARDS in ICU patients: a retrospective cohort study
Source: Open Med (Wars). 2026 Mar 18;21(1):20261401. doi: 10.1515/med-2026-1401 (PMC12995391; doi:10.1515/med-2026-1401)
Supplement: Supplementary file 1 — Supplementary Material [file j_med-2026-1401_suppl_001.docx]

**Supplementary Table 1. Results of the Fine-Gray competing risks model analysis considering death as a competing event**

| Variable | Total Number | Number of Events | sHR (95% CI) | *P* |
| --- | --- | --- | --- | --- |
| Age，years | 482 | 108 | 1.05 (1.02, 1.07) | <0.001 |
| Pneumonia (yes) | 153 | 50 | 1.78 (1.21, 2.60) | 0.001 |
| Sepsis (yes) | 237 | 69 | 1.59 (1.09, 2.33) | 0.02 |
| SOFA rating | 482 | 108 | 0.92 (0.84, 1.01) | 0.072 |
| LYM, ×10⁹/L | 482 | 108 | 0.81 (0.47, 1.40) | 0.450 |
| EOS Status (EOS(-)) | 202 | 60 | 1.44 (0.91, 2.29) | 0.121 |
| BMI，kg/m^2^ | 482 | 108 | 0.94 (0.89, 1.00) | 0.083 |
| Diabetes (yes) | 105 | 37 | 1.46 (0.97, 2.22) | 0.102 |

sHR, Subdistribution Hazard Ratio; CI, Confidence Interval.

The Fine-Gray competing risks model was employed, treating in-hospital death as a competing event for ARDS occurrence.

This model estimates the “subdistribution” hazard ratio for ARDS occurrence, assuming patients did not exit observation prematurely due to the competing event (death).
